# Mapping resistance to powdery mildew in barley reveals a large-effect nonhost resistance QTL

**DOI:** 10.1007/s00122-018-3055-0

**Published:** 2018-01-25

**Authors:** Cynara C. T. Romero, Jasper P. Vermeulen, Anton Vels, Axel Himmelbach, Martin Mascher, Rients E. Niks

**Affiliations:** 1 Plant Breeding, Wageningen University & Research, PO Box 386, 6700 AJ Wageningen, The Netherlands; 2 Leibniz Institute of Plant Genetics and Crop Plant Research (IPK) Gatersleben, 06466 Seeland, Germany

## Abstract

Resistance factors against non-adapted powdery mildews were mapped in barley. Some QTLs seem effective only to non-adapted mildews, while others also play a role in defense against the adapted form.

The durability and effectiveness of nonhost resistance suggests promising practical applications for crop breeding, relying upon elucidation of key aspects of this type of resistance. We investigated which genetic factors determine the nonhost status of barley (*Hordeum vulgare* L.) to powdery mildews (*Blumeria graminis*). We set out to verify whether genes involved in nonhost resistance have a wide effectiveness spectrum, and whether nonhost resistance genes confer resistance to the barley adapted powdery mildew. Two barley lines, SusBgt_SC_ and SusBgt_DC_, with some susceptibility to the wheat powdery mildew *B. graminis* f.sp. *tritici* (*Bgt*) were crossed with cv Vada to generate two mapping populations. Each population was assessed for level of infection against four *B. graminis* ff.spp, and QTL mapping analyses were performed. Our results demonstrate polygenic inheritance for nonhost resistance, with some QTLs effective only to non-adapted mildews, while others play a role against adapted and non-adapted forms. Histology analyses of nonhost interaction show that most penetration attempts are stopped in association with papillae, and also suggest independent layers of defence at haustorium establishment and conidiophore formation. Nonhost resistance of barley to powdery mildew relies mostly on non-hypersensitive mechanisms. A large-effect nonhost resistance QTL mapped to a 1.4 cM interval is suitable for map-based cloning.

**Electronic supplementary material:**

The online version of this article (10.1007/s00122-018-3055-0) contains supplementary material, which is available to authorized users.

## Introduction

Plants are exposed to infinite microorganisms during their lifespan, many of which are potentially harmful. The evolution of a sophisticated and dynamic immune system has enabled plants to protect themselves against most infectious microorganisms. For a pathogen to be successful in infecting a host plant, it must be adapted to overcome several layers of defence (Jones and Dangl 2006; Nurnberger and Lipka 2005; Thordal-Christensen 2003). The most common outcome of infection attempts by potential pathogens on plants is failure, making by far most plant species nonhosts. Nonhost resistance has been defined as immunity of an entire plant species against all races of a particular non-adapted pathogen (Heath 2000; Lipka et al. 2010; Mysore and Ryu 2004). The typical durability and effectiveness of nonhost resistance suggests promising practical applications in breeding programs (Heath 2000; Lee et al. 2016; Niks 1987; Nurnberger and Lipka 2005). Two models were proposed in the last decade to explain nonhost resistance (Jones and Dangl 2006; Schweizer 2007). According to the first model, plant cell surface receptors (known as pattern-recognition receptors, PRRs) perceive pathogen-associated molecular patterns (PAMPs, also referred to as microbial-associated molecular patterns, MAMPs) or endogenous damage-associated molecular patterns (DAMPs) and trigger the first layer of defence response known as PAMP-triggered immunity (PTI). Non-adapted pathogens fail to suppress PTI because their effector repertoire is not adapted to nonhost plant targets to undermine defence. The second model presupposes that nonhost resistance is the result of active participation of intracellular receptors, mainly nucleotide-binding—leucine-rich repeat (NB-LRR) proteins encoded by resistance (*R*) genes. Effector molecules released by the pathogen to undermine PTI would be perceived by NB-LRRs, triggering a second layer of defence known as effector-triggered immunity (ETI) (Stam et al. 2014; Zhang et al. 2013). There are several examples of the participation of PTI and ETI on nonhost resistance [reviewed in Lee et al. (2017)], and, although distinction between PAMPs and effectors may not be strict (Thomma et al. 2011), it is still an issue whether nonhost resistance relies mainly on PTI or on ETI. Schulze-Lefert and Panstruga (2011) hypothesized that, for cases where host and nonhost plant species are phylogenetically closely related, the contribution of ETI to nonhost resistance would be relatively higher than that of PTI.

Despite the undeniable practical relevance of nonhost resistance, the genetic mechanisms governing the (non)host status of a plant to a potential pathogen species are yet to be elucidated. It is known that basal resistance, defined as the “resistance activated by virulent pathogens on susceptible hosts” (Jones and Dangl 2006), and nonhost resistance share several aspects (Gill et al. 2015; Niks and Marcel 2009). Many studies using reverse genetics approaches have identified genes involved in basic plant metabolism as contributing to nonhost resistance (Lee et al. 2016). Such genes, mostly components of general plant defence mechanisms, are widely conserved among plant species, and therefore, their identification by mutagenesis or transcriptomics is not sufficient to explain the nonhost status of a plant (Niks 2014; Niks and Marcel 2009). Inheritance and mapping studies are, for this reason, necessary to reveal which genes determine host–nonhost status to a potential pathogen. The fundamental problem in studying the inheritance of nonhost resistance is its dependence on host × nonhost interspecific crosses, which are usually not interfertile (Niks and Marcel 2009). It was proposed that studying the genetics of the resistance in plants that have an intermediate status between host and nonhost could provide useful insights (Atienza et al. 2004; Zhang et al. 1994). Some plant species may be regarded as ‘near-nonhosts’ or ‘marginal-hosts’ with a few accessions being somewhat susceptible to a normally non-adapted pathogen (Niks 1987). This susceptibility may be true solely during seedling stage and/or when under high inoculum density.

Barley (*Hordeum vulgare* L.) is a near-nonhost to many non-adapted pathogens of cereals and grasses, including rust and powdery mildew fungi. Aghnoum and Niks (2010) tested 439 barley accessions for resistance to the non-adapted *Blumeria graminis* f.sp. *tritici* (*Bgt*), the wheat powdery mildew fungus. The great majority of the accessions were immune, but at least six showed a low degree of susceptibility. Four of those were selected to be inter-crossed and to develop two lines with increased susceptibility to *Bgt* at seedling stage. These lines, named SusBgt_SC_ and SusBgt_DC_, allowed a relatively high rate of haustorium formation by *Bgt* and three other non-adapted *B. graminis* forms. In barley, nonhost resistance to powdery mildews is typically due to formation of localized cell wall reinforcements, called papillae, preventing haustorium formation (Trujillo et al. 2004). Papilla formation is also a main feature of basal host resistance, as in barley with *mlo* resistance or with high gene dose of quantitative resistance to *B. graminis* f.sp. *hordei* (*Bgh*) (Aghnoum et al. 2010; Niks and Rubiales 2002). Although *Bgt* is not able to form as large colonies on barley leaves as it would on its host, the germlings that are able to penetrate the cell and establish a haustorium can grow enough mycelium to form micro-colonies: tiny white dots on the epidermal layer of young leaves. Micro-colonies depend mostly on one successful haustorium, or on several haustoria in one successfully colonized plant cell. Further attempts to penetrate additional plant cells were generally not successful (Aghnoum and Niks 2010).

The main goal of our research was to map the gene(s) underlying nonhost and basal host resistance in barley against four ff.spp. of *B. graminis* (three non-adapted and the adapted form). We determined whether genes involved in nonhost resistance may have a wide spectrum of effectiveness, with the same gene(s) having effect to multiple powdery mildew forms, and whether nonhost resistance genes may also confer resistance to the barley-adapted *Bgh*. Two mapping populations were developed by crossing the SusBgt lines with the barley cv Vada: Vada × SusBgt_SC_ and Vada × SusBgt_DC_. We developed a high-density genetic map for each SusBgt population, using the genotyping-by-sequencing technology (Elshire et al. 2011; Poland et al. 2012). The QTL mapping results bring us one step further in the identification of genes responsible for the specificity of (non)host status.

## Materials and methods

### Plant material and DNA extraction

Two barley lines selected for relatively high susceptibility to the non-adapted mildew *Bgt* (SusBgt_SC_ and SusBgt_DC_) were crossed with cv Vada to develop two Recombinant Inbred Line (RIL) mapping populations. The Vada × SusBgt_SC_ (VxS_SC_) population consists of 110 RILs (104 RILs in F_7_ generation and 6 in F_8_) and the Vada × SusBgt_DC_ (VxS_DC_) population consists of 115 RILs (14 RILs in F_6_ generation, 8 in F_7_, 86 in F_8_, and 7 in F_9_). Genomic DNA of the RILs from both populations was extracted from leaf tissue of 16-day-old seedlings (one seedling per RIL), using a modified version of the CTAB method (Fulton et al. 1995). DNA samples were RNase-treated and column-cleaned using the Quiagen DNeasy Plant Midi kit. DNA concentrations were quantified using the QubitBR kit (Thermofisher Scientific) and diluted to a final concentration of 20–25 ng/µL. The integrity of DNA samples was confirmed on a 0.8% agarose gel with 1% ethidium bromide.

### Genotyping and genetic map construction

Both mapping populations were genotyped using the Genotyping-by-sequencing (GBS) approach (Elshire et al. 2011) following a two-enzyme protocol (Poland et al. 2012) essentially as described previously (Wendler et al. 2014). For sequencing-by-synthesis (single read, 1 × 100 cycles), the Illumina HiSeq2500 device (IPK Gatersleben, Germany) was employed (Wendler et al. 2014). Illumina adapters were trimmed from the raw reads using Cutadapt version 1.8.1 (Martin 2011). Trimmed reads were aligned to the whole-genome shotgun assembly of barley cv Morex (International Barley Genome Sequencing Consortium 2012) using BWA-MEM version 0.7.12 (Li 2013). After conversion to BAM format with SAMtools (Li et al. 2009), the resulting alignments were sorted and indexed with Novosort (http://www.novocraft.com/products/novosort/). SNP calling was performed with SAMtools version 1.3 (Li 2011) using the commands ‘samtools mpileup –DV’ and ‘bcftools call –c –f GQ’. The resulting VCF file was filtered with the AWK script gen_call.awk provided by Mascher et al. (2013b). Only SNPs with a minimum quality (QUAL) of 40 were considered. Genotype calls were set to missing if their coverage was below 2 (4) and their quality score (GQ) below 5 (10) for homozygous (heterozygous) calls. Genetic maps were calculated separately for both populations. Only SNPs with a minor allele frequency of at least 30% and missing rate below 10% were considered for map construction. Linkage maps were built with MSTMAP (Wu et al. 2008) using the population type ‘RIL8’ and a cut-off *P* value of 10^−12^. Correctness of the maps was assessed by comparison to the POPSEQ reference map (Mascher et al. 2013a) using R scripts (R Core Team 2016).

A set of markers homogeneously distributed along the chromosomes at distances of ~ 3 cM was extracted from the SNP matrices, with the condition that they were polymorphic for both populations—that would facilitate later comparison of QTL positions among populations. Selected markers were used to build a skeletal map for each mapping population for QTL mapping analysis.

### Inoculum material and inoculation trials

Four isolates, each belonging to a different *forma specialis* (f.sp.) of *Blumeria graminis* were tested: the powdery mildew fungus of wheat, *B. graminis* f.sp. *tritici* (*Bgt*, Swiss field isolate FAL92315), two isolates collected from wild grasses (*Hordeum murinum* and *Hordeum secalinum*) near Wageningen-NL, and referred to in this paper as: *B. graminis* f.sp. *hordei*-*murini* (*Bghm*), and *B. graminis* f.sp. *hordei*-*secalini* (*Bghs*), respectively; the fourth f.sp. was the adapted powdery mildew of barley, *B. graminis* f.sp. *hordei* (*Bgh*, collected in Wageningen). The mildew isolates were continuously propagated on their respective host plants (for wheat: cv Vivant; for barley: cv Manchuria).

**Fig. 1:**
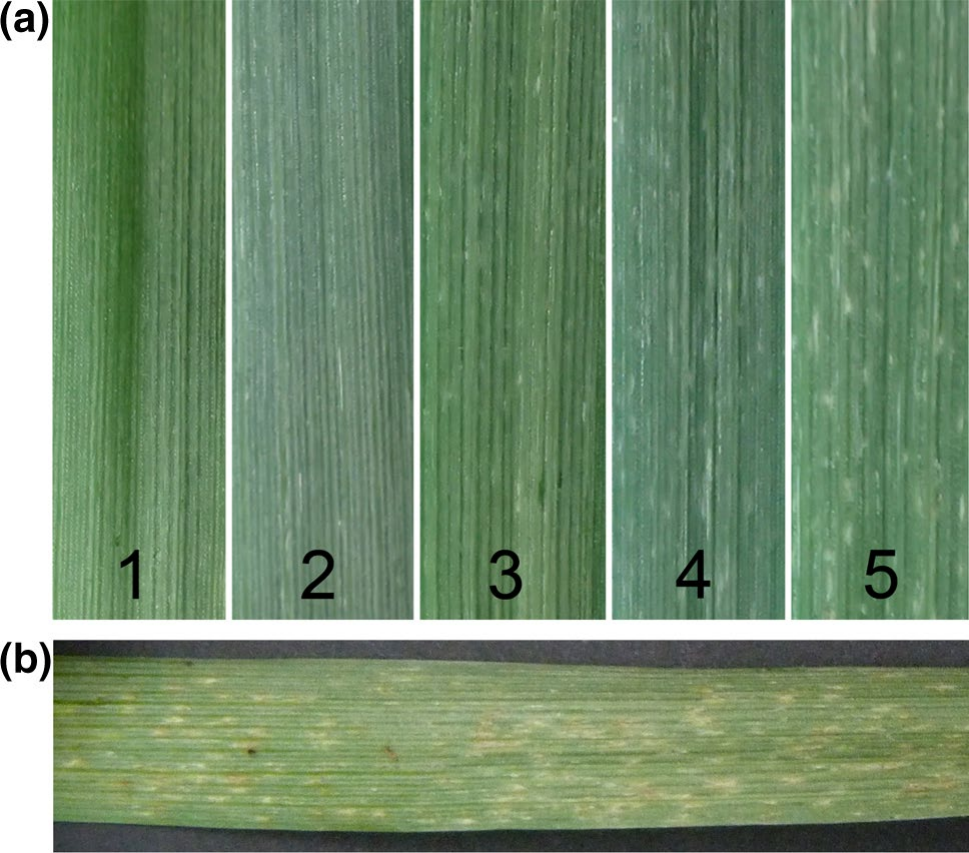
**a** Illustration of the relative scale values used to assess the degree of micro-colony development on the surface of barley (*Hordeum vulgare*) leaves 7 days after inoculation with *Blumeria graminis* f.sp. *tritici* (*Bgt*) or f.sp. *hordei*-*murini* (*Bghm*); **b** barley line SC-28 8 days after inoculation with *Blumeria graminis* f.sp. *tritici* (*Bgt*), showing mild necrotic reaction phenotype

Each population was phenotyped for the level of infection in two consecutive experiments, with two seedlings/RIL per experiment. The whole set of RILs was grown in boxes (40 × 60 cm), together with the parent Vada and both SusBgt_SC_ and SusBgt_DC_ lines as references. Also the host plants (either wheat cv Vivant, *H. murinum* or *H. secalinum*, depending on the inoculation experiment) were included in the trays to verify the viability of the inoculum. Compost soil was used as a substrate. The seedlings grew up in a controlled growth chamber (18–20 °C day time, 15 °C night time, 40–60% relative humidity, 16 h photoperiod) until they were *c*. 13 days old. The first leaf of each seedling was pinned horizontally to the substrate with the adaxial side up, using metal pins; remaining emerging leaves were removed. Inoculations were performed in a settling box (100 cm × 120 cm × 87 cm height), where the entire population was placed to be inoculated at once. Fresh conidia from heavily sporulating host leaf segments were blown into the settling box using compressed air, until the aimed density was reached. For the non-adapted forms (*Bgt*, *Bghm* and *Bghs*), the density was 20–30 conidia/mm^2^; for the adapted pathogen (*Bgh*), around 5 conidia/mm^2^. Metal pins were kept on the leaves until the next day. Inoculated seedlings were transferred to a second compartment (same conditions as previous one) where they were kept until the moment of evaluation. Macroscopic evaluation occurred 7 days after inoculation (dai) with the non-adapted mildew, when seedlings were assessed for level of infection. Non-adapted fungi can only grow enough to form micro-colonies, visible as small white spots over the surface of the leaf. A relative scale was set, in which the score of each RIL was always given in comparison to the references Vada (resistant, no micro-colony formation; score 1) and SusBgt lines (susceptible, high degree of micro-colony formation; score 5; Fig. 1a). Some RILs showed more fungal growth than the respective SusBgt parent, and therefore, were given a score higher than 5. Phenotyping of the *Bgh*-inoculated plants occurred 4 dai, by assessing infection frequency (number of colonies formed in a 2 × 1 cm^2^ area) using a metal frame with a rectangular opening of 1 cm^2^ and a magnifying glass. Seven days after inoculation with *Bgh*, the presence of necrotic reaction was also assessed, on a scale of 1–4 (1: no necrotic reaction observed; 4: highest necrosis reaction observed in the population).

### Microscopic evaluation of barley *Bgt* interaction

Seven RILs from the VxS_SC_ population and 13 RILs from the VxS_DC_ population scoring higher than 3 during the macroscopic phenotyping with *Bgt* were sampled to assess microscopically the number of established micro-colonies/cm^2^ and the conidiation rate (percentage of micro-colonies that produced at least one conidiophore). The parental lines were included in the microscopic analysis to serve as references.

Leaf segments of c. 4 cm were transferred to tubes containing a solution of acetic acid–ethanol (1:3 v/v) to be cleared for at least 1 day. For staining, segments of *c*. 1 cm were cut and immersed for 25 min in a solution of 15% trichloroacetic acid and 0.6% Coomassie Brilliant Blue in 99% methanol (1:1 v/v) (Wolf and Fric 1981). Leaf segments were then transferred to a solution of acetic acid: glycerol: Milli-Q water (5:20:75) for 5–10 min to remove the excess of dye. Microscope slides were prepared by embedding the stained leaf segments in 100% glycerol, with the adaxial side up. Slides were screened using bright field microscopy with a total 100× magnification under a white light microscope. Germlings showing secondary elongating hyphae were considered as established (here called micro-colonies). For each sample, the number of micro-colonies was counted and expressed in micro-colonies/cm^2^. For each barley line, segments of two leaves were assessed per inoculation experiment. Statistical analyses were performed using GenStat 18th edition (Hemel Hempstead, UK). An ANOVA followed by a Fisher’s unprotected LSD (*P* < 0.05) was performed to test for significant differences in the rates of establishment and conidiation.

### QTL mapping

QTL analyses for both mapping populations were performed using MapQTL 6 software (Van Ooijen 2009). QTL mapping files are provided as supplementary material (Online Resource 1). The skeletal maps of VxS_SC_ and VxS_DC_ contained 354 and 372 markers, respectively. QTL mapping was performed independently for the two replicate experiments and for the average of both. The mapping analysis was done in three steps. First, an interval mapping (IM) was performed using a mapping step size of 5 cM. A LOD threshold of 2.9 was set (estimated with a permutation test at 1000 permutations, using a significance level of *P* < 0.05) to declare QTLs. The identified QTL peak markers were chosen as cofactors for the subsequent mapping steps, multiple-QTL mapping (MQM) and restricted multiple-QTL mapping (rMQM) (Jansen 1993; Jansen and Stam 1994).

Graphical maps of both populations were constructed using MapChart v2.3 (Voorrips 2002), to indicate the regions where QTLs were found. The averages of macroscopic disease scores for QTL allele combinations were compared and tested for significant differences with an ANOVA following a Fishers’ unprotected LSD (*P* < 0.05) using GenStat 18th edition (Hemel Hempstead, UK).

### Conidia viability test

The viability of *Bgt* conidia produced on barley plants was tested for the ability to re-infect its natural host, wheat. For this trial, SusBgt_SC_, SusBgt_DC_, Vada and one RIL of each population were selected: SC-45 and DC-02. Both RILs had an average macroscopic score slightly higher than their susceptible parent. Three seeds per genotype were sown, and 12 days later seedling leaves were inoculated with *Bgt* to a density of 22 conidia/mm^2^, following the same inoculation procedure previously described. Ten days after inoculation, the infected barley leaves were detached and rubbed against the leaves of 11 days old wheat cv. Vivant plants. Each of the three barley seedlings per genotype was gently rubbed against the first and second leaves of an individual wheat plant, only in areas delimited by a marker pen. Wheat seedlings not treated with any barley leaf were also added to the experiment as negative controls. The growth of *Bgt* colonies on wheat seedlings was assessed 5 days later.

## Results

### Phenotyping of the resistance to non-adapted *Blumeria* forms

The two mapping populations were evaluated macroscopically for degree of micro-colony formation by the non-adapted forms *Bgt*, *Bghm* and *Bghs*. For *Bghs* it was not possible to observe any micro-colony on the parents nor on a subset of 50 random RILs, even 14 dai (Online Resource 2a). Samples of leaves from the parental lines inoculated with *Bghs* were examined under the microscope. We observed 2.2 and 4.5 micro-colonies/cm^2^ for SusBgt_SC_ and SusBgt_DC_, respectively, which was apparently too low to result in macroscopically visible infection.

For *Bgt*, RILs were scored on a scale of 1–5, having the phenotypes of the parental lines as references (Fig. 1a, Online Resource 2b). This phenotyping method proved reliable, as indicated by the high correlation of scores between inoculation experiments (Online Resource 3). The majority of RILs showed no macroscopic symptom to *Bgt* infection, and were given the lowest score ‘1’ (Online Resource 4a–b). The highest scores were assigned to lines with a similar level of fungal growth as on the SusBgt parent. A one-way ANOVA followed by Fisher’s unprotected LSD (*P* < 0.01) on the top 50 RILs with highest scores in each population showed that 7 out of 110 (6.36%) in the VxS_SC_, and 10 out of 115 (8.69%) in the VxS_DC_ population scored not significantly lower than the respective SusBgt parent. Scores for 41 out of 110 RILs for VxS_SC_ and 41 out of 115 RILs for the VxS_DC_ population were continuously distributed between 1.5 and 5.

The distribution of the macroscopic disease scores for *Bghm* was similar to those for *Bgt*: more than 75% of RILs in both populations scored lower than ‘2’, and only a small number of RILs had scores above ‘4’ (Online Resource 4c–d, Online Resource 3). Average infection scores for *Bgt* and *Bghm* were highly correlated (*r* > 0.7 for both populations). The shape of the frequency distributions, with the vast majority of RILs showing a resistant phenotype, suggests that several genes are involved in the nonhost resistance. The very skewed frequency distributions suggests that resistance alleles at one of the loci already results in a substantial level of resistance. A limited hypersensitive reaction (HR) was observed in association with micro-colonies development, in some RILs (Fig. 1b).

### Genetic map construction and QTL mapping

The SusBgt mapping populations were genotyped using the Genotyping-by-sequencing (GBS) approach (Elshire et al. 2011) following a two-enzyme protocol (Poland et al. 2012; Wendler et al. 2014). We obtained on average 1.7 million reads per sample (min: 0.5 million; max: 14.8 million). Read mapping against the whole-genome shotgun assembly of barley cv Morex (International Barley Genome Sequencing Consortium 2012), SNP calling and linkage map construction were performed following a previously published pipeline (Mascher et al. 2013b).

The high-density genetic maps contained a total of 6966 (VxS_SC_) and 7422 (VxS_DC_) SNP markers (Online Resource 5). The largest gap between two adjacent loci was 6.21 cM in the VxS_SC_ population on chromosome 1H (from 100.2 to 106.4 cM) and 6.42 cM on chromosome 2H (from 68.8 to 75.3 cM) in VxS_DC_. Total genetic lengths of the linkage maps were 1007 cM for VxS_SC_ and 1023 cM for VxS_DC_.

**Fig. 2:**
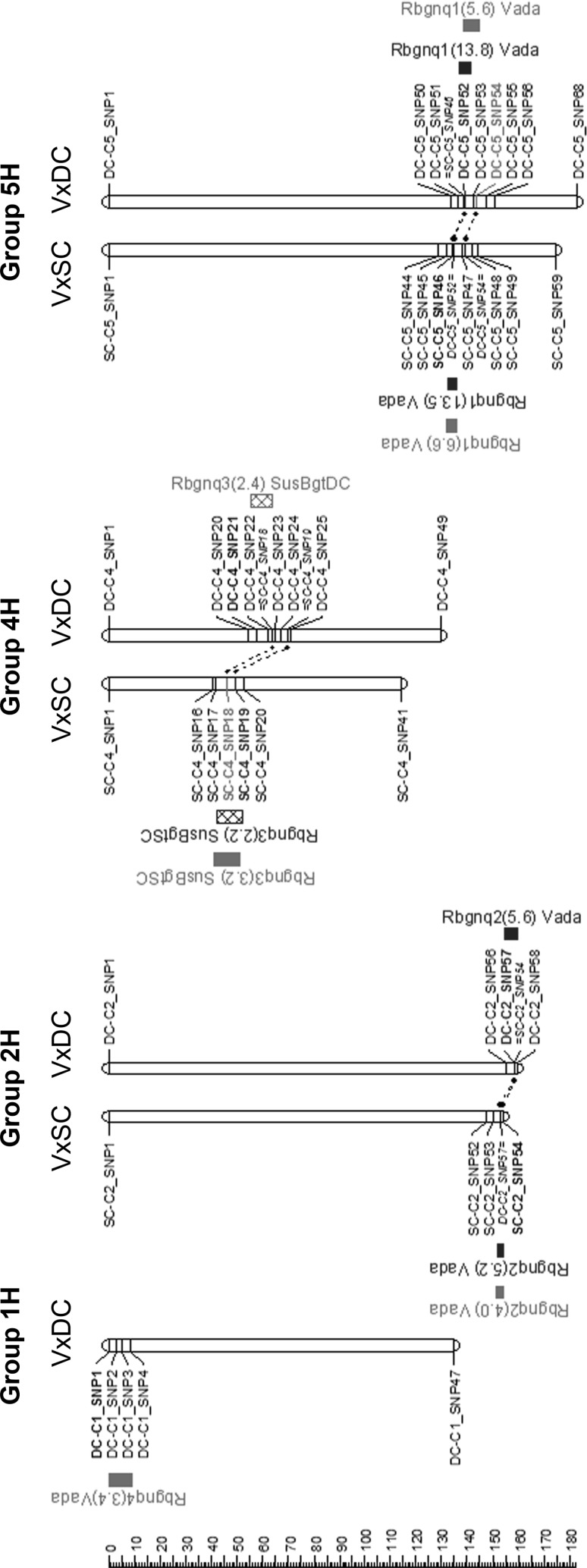
Localization of QTLs for nonhost resistance to powdery mildew mapped in the Vada × SusBgt_SC_ (VxSC) and Vada × SusBgt_DC_ (VxDC) mapping populations. Bars along each linkage group represent the LOD-1 interval of QTLs mapped for resistance to different ff.spp., indicated in colours: blue = *Blumeria graminis* f.sp. *tritici* (*Bgt*); green = f.sp. *hordei*-*murini* (*Bghm*). Shaded bars represent QTLs below the LOD threshold. Label to each QTL region mentions name of the QTL, its LOD score and the name of the parent contributing the resistance allele. For each linkage group, only the first and last markers of the skeletal map are represented, plus the markers at the QTL-containing regions. Linkage groups that did not contain significant QTLs were omitted from this figure. The ruler on the left indicates the distance in cM

Two RILs were excluded from the QTL mapping analysis due to a high percentage of missing genotyping data: DC-26 and DC-101. QTLs mapped in this study for resistance to non-adapted mildews were named ‘*Rbgnq*’ (acronym for Resistance to *Blumeria* *graminis* nonhost quantitative) and followed by a number, according to the order in which they were mapped. In total, four chromosome regions were associated with nonhost resistance. QTL mapping results based on macroscopic disease scores were similar for *Bgt* and *Bghm* (Fig. 2). Peak markers of QTLs mapped in one population were in general overlapping with the LOD-1 region of a QTL mapped on the other population, and therefore, received the same name (Table 1). The two largest effect QTLs (*Rbgnq1* and *Rbgnq2*) are effective to both non-adapted forms. *Rbgnq1*, on linkage group 5H, had the highest LOD scores and estimated additive effects; it appeared consistently over the inoculation experiments in both SusBgt populations and is a major-effect QTL for nonhost resistance to powdery mildew. Another QTL, *Rbgnq3*, has the resistance allele contributed by the susceptible parent. *Rbgnq3* was sometimes mapped with a LOD score slightly below threshold, but the data were still included in Table 1 because the LOD score for resistance to *Bghm* in the VxS_SC_ population was above the threshold. *Rbgnq4*, located near the telomere of the short arm of chromosome 1H was mapped only in the VxS_DC_ population for resistance to *Bghm*.

**Table 1:** Summary of QTL mapping in the SusBgt mapping populations (Vada × SusBgt_SC_, VxS_SC_; and Vada × SusBgt_DC_, VxS_DC_) for nonhost resistance to *Blumeria graminis* f.sp. *tritici* (*Bgt*) and f.sp. *hordei*-*murini* (*Bghm*) and basal resistance to *B. graminis* f.sp*. hordei*

Population	f.sp.	Trait^a^	QTL name^b^	Chr^c^	Peak marker	Position (cM)	LOD-1 interval (cM)	LOD^d^	% Expl^e^	Additive^f^	Donor resistance	Mapped for other f.sp.^g^
VxS_SC_	*Bgt*	Micro-colonies	*Rbgnq1* *	5H	SC_C5-SNP46	134.2	132.9–135.8	13.5	41.8	− 0.73	Vada	*Bghm*
	*Bgt*	Micro-colonies	*Rbgnq2* *	2H	SC_C2-SNP54	154.0	152.1–154.0	5.2	13.3	− 0.41	Vada	*Bghm, Bgh* (= *Rbghq1*)
	*Bgt*	Micro-colonies	*Rbgnq3*	4H	SC_C4-SNP19	49.5	42.2–52.2	2.2	4.9	0.24	SusBgt_SC_	*Bghm*
	*Bghm*	Micro-colonies	*Rbgnq1* *	5H	SC_C5-SNP46	134.2	132.3–136.0	6.6	20.7	− 0.40	Vada	*Bgt*
	*Bghm*	Micro-colonies	*Rbgnq2*	2H	SC_C2-SNP54	154.0	151.6–154.0	4.0	11.9	− 0.29	Vada	*Bgt*, *Bgh* (= *Rbghq1*)
	*Bghm*	Micro-colonies	*Rbgnq3*	4H	SC_C4-SNP18	46.0	41.2–50.8	3.2	9.3	0.25	SusBgt_SC_	*Bgt*
	*Bgh*	*IF*	*Rbghq1*	2H	SC-C2_SNP54	154.0	153.2–154.0	11.6	35.1	− 4.794	Vada	*Bgt*, *Bghm* (= *Rbgnq2*)
	*Bgh*	*IF*	*Rbghq3*	6H	SC-C6_SNP40	116.8	111.5–119.2	2.9	7.1	− 2.208	Vada	No
	*Bgh*	*Nec*	Rbghq1*	2H	SC-C2_SNP54	154.0	153.4–154.0	26.0	68	1.084	Vada	*Bgt*, *Bghm* (= *Rbgnq2*)
VxS_DC_	*Bgt*	Micro-colonies	*Rbgnq1* *	5H	DC_C5-SNP52	139.2	137.1–141.7	13.8	37.7	− 0.70	Vada	*Bghm*
	*Bgt*	Micro-colonies	*Rbgnq2* *	2H	DC_C2-SNP57	158.3	154.9–159.7	5.6	12.9	− 0.40	Vada	*Bgh* (= *Rbghq1*)
	*Bghm*	Micro-colonies	*Rbgnq1*	5H	DC_C5-SNP54	143.4	139.0 –144.6	5.6	17.0	− 0.42	Vada	*Bgt*
	*Bghm*	Micro-colonies	Rbgnq3*	4H	DC_C4-SNP21	57.4	55.5–63.6	2.4	6.3	0.24	SusBgt_DC_	No
	*Bghm*	Micro-colonies	*Rbgnq4*	1H	DC_C1-SNP1	0	0–9.2	3.4	9.8	− 0.31	Vada	No
	*Bgh*	*IF*	Rbghq1*	2H	DC-C2_SNP58	159.7	159–159.7	8.3	20.6	− 2.738	Vada	*Bgt* (= *Rbgnq2*)
	*Bgh*	*IF*	*Rbghq2*	7H	DC-C7_SNP2	3.2	0–4.0	11.8	31.6	3.406	SusBgt_DC_	No
	*Bgh*	*Nec*	Rbghq1*	2H	DC-C2_SNP58	159.7	158.2–159.7	4.0	9.8	0.38	Vada	*Bgt* (= *Rbgnq2*)
	*Bgh*	*Nec*	*Rbghq2*	7H	DC-C7_SNP1	0	0–0.3	13.2	39.4	− 0.77	SusBgt_DC_	No

Mapping is based on results from the average of two inoculation experiments

^a^For nonhost interactions, the macroscopic infection score (‘micro-colonies’, data correspond to the average of two inoculation experiments) was assessed; for basal resistance, both ‘IF’ (Infection frequency, data from the average of two inoculation experiments) and ‘Nec’ (necrosis phenotype, based on the results of the second inoculation experiment) were assessed

^b^The QTLs mapped for nonhost resistance (Bgt and Bghm) are named ‘*Rbgnq*’ and those mapped for basal resistance (*Bgh*) were named ‘*Rbghq,*’, in both cases followed by a number based on the order and relevance in which they were mapped. The QTL name is followed by an asterisk (*) if its peak marker is located within the LOD-1 region of the QTL that was mapped for the same f.sp. on the second population; underlined QTLs were mapped consistently over the two inoculation experiments

^c^The chromosome (linkage group) in which the QTL was mapped

^d^The LOD-score of the QTL

^e^The proportion of phenotypic variance explained by the QTL

^f^The effect of having one allele from Vada on the macroscopic infection score, infection frequency or necrosis score

^g^Indicates whether the QTL was also mapped for other f.sp.in the same mapping population

To look for possible interaction effects of QTLs on the macroscopic infection scores, we grouped the RILs according to the alleles of the nonhost resistance QTLs (Table 2; refer to Online Resource 6 for similar results on the VxS_DC_ population). In general, RILs only show high susceptibility scores when all resistance alleles are absent. Because of the high contribution of *Rbgnq1* to the phenotype, RILs carrying the resistance allele for this locus show the resistant phenotype irrespective of the background QTLs. In the absence of *Rbgnq1*, a similar resistant phenotype can be achieved if the resistance allele of the other two QTLs are both present. A couple of VxS_SC_ RILs carrying the Vada allele of the peak marker of *Rbgnq1* showed an unexpected high average score for *Bgt*, so we retrieved from the original high-density genetic map additional markers at this locus. Based on recombination points located in between the markers used for QTL mapping, it was possible to narrow-down the QTL interval to a window of 1.4 cM (Online Resource 7). The flanking markers were aligned to the map-based reference genome of cv Morex (Mascher et al. 2017) and delimit an interval containing 188 (VxS_SC_) and 104 (VxS_DC_) predicted genes (Online Resource 7).

**Table 2:** Average macroscopic infection scores for VxS_SC_ recombinant inbred lines (RILs) grouped according to presence (+) or absence (−) of the resistance allele of QTLs mapped for *Blumeria graminis* f.sp. *tritici* (*Bgt*) and f.sp. *hordei*-*murini* (*Bghm*)

*Rbgnq1* (V)	*Rbgnq2* (V)	*Rbgnq3* (SC)	Number of RILs^a^	*Bgt*		*Bghm*	
+	+	+	11	1.1	a	1.1	a
+	+	−	10	1.1	a	1.1	a
+	−	+	19	1.1	a	1.2	a
+	−	−	14	1.1	a	1.3	ab
−	+	+	20	1.6	ab	1.1	a
−	+	−	14	2.1	b	2.0	bc
−	−	+	8	3.3	c	2.3	cd
−	−	−	8	4.3	d	3.1	d

Corresponding resistance alleles of each QTL are into brackets (V = Vada; SC = SusBgt_SC_). Values in each column that share the same letter are not significantly different (*P* < 0.05)

^a^Total number of RILs analysed: 104 for *Bgt* and 105 for *Bghm*. The number of RILs in each group differed slightly (± 2 RILs) between *Bgt* and *Bghm* scores because the peak marker of *Rbgnq3* was different for the two ff.spp. or because of missing phenotyping data. Four RILs were excluded from the analysis because there was a recombination point close to the peak marker of *Rbgnq1*

### Developing near-isogenic lines for a nonhost resistance QTL using RIL DC-04

A considerable difference in phenotypic scores was noticed for RIL DC-04 for the two *Bgt* inoculation experiments. This RIL was in F_8_ and hence, harvested from a single F_7_ plant. During the first inoculation DC-04 was given the maximum score of 5 (susceptible), while for the second inoculation it was given the minimum score of 1 (resistant). A third inoculation was set up for this line, and among the three DC-04 seedlings, one was susceptible and two were resistant (Online Resource 8). Probability for a marker or gene in F_7_ to be heterozygous, and hence segregating in F_8_, is (0.5)^6^, which is 1.6%. In a set of 115 RILs it is, therefore, expected to find about one or two such segregating RILs for a particular locus. We found a segment of ~ 8 cM on chromosome 5H segregating for the region of *Rbgnq1* in this particular RIL, explaining the segregation in phenotype. RIL DC-04, therefore, is a heterogeneous inbred family (HIF), from which a pair of near-isogenic lines is being developed, as proposed by Tuinstra et al. (1997).

### Microscopic evaluation of *Bgt*-infected lines

**Fig. 3:**
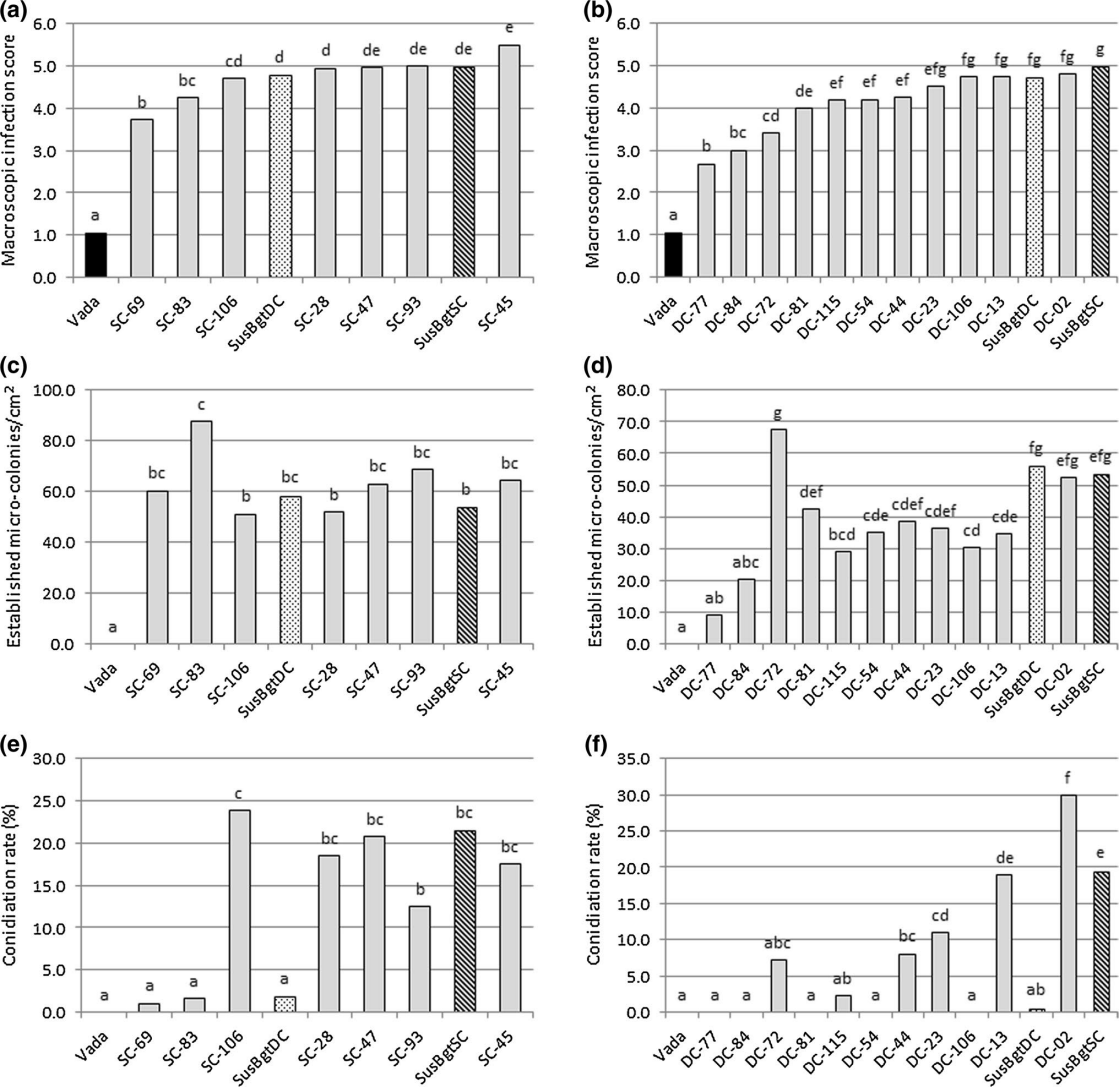
Microscopic data from the interaction of *Blumeria graminis* f.sp. *tritici* (*Bgt*) with a subset of recombinant inbred lines (RILs) from the VxS_SC_ and VxS_DC_ mapping populations, including the parents. The bars represent average data of two replicate experiments, with two leaf segments per experiment. Parental lines are represented by black bars for Vada, grey checkered bars for SusBgt_DC_, and diagonally hatched bars for SusBgt_SC_. **a**, **b** Macroscopic infection scores 7 days after inoculation with *Bgt*. **c**, **d** Number of established micro-colonies/cm^2^ counted under the microscope 8 days after inoculation with *Bgt*. **e**, **f** Conidiation rate: percentage of established micro-colonies that formed conidia 8 dai with *Bgt*. Within each chart, bars sharing the same letter are not significantly different (*P* < 0.05)

**Fig. 4:**
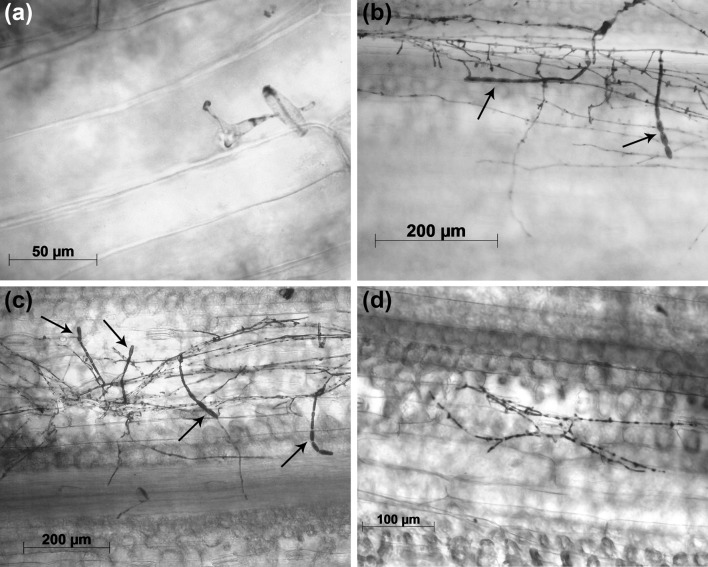
Infection units of *Blumeria graminis* f.sp. *tritici* (*Bgt*) on barley (*Hordeum vulgare*) plants, 8 days after inoculation. Conidiophores are indicated with an arrow. **a** A stopped penetration attempt including papilla formation on Vada. **b** An established micro-colony with conidiophores on the susceptible parent SusBgt_SC_. **c** Established micro-colony with conidiophores on SC-45. **d** Established micro-colony without conidia on DC-106

Seven RILs from the VxS_SC_ population and 11 RILs from the VxS_DC_ population were selected for microscopic analysis and sampled from the same experiments for which the macroscopic scores were recorded. As expected, most of the infection attempts, in all RILs, were stopped in association with papilla formation and Vada did not show any established micro-colonies (Figs. 3c, d, 4a). For both SusBgt parents, a similar number of around 50–60 established micro-colonies/cm^2^ was observed (Fig. 3c, d), from the total of c. 2550 conidia inoculated per cm^2^ area, implying that at most 3% of the applied spores succeeded in establishing haustoria in the barley epidermis. Some variation was observed in the number of established micro-colonies/cm^2^ between RILs with similar macroscopic infection scores (Fig. 3a–d). This situation is well illustrated by RILs SC-83 and SC-106: their macroscopic scores were 4.3 and 4.7, respectively, but the number of established micro-colonies/cm^2^ differed significantly from 87.5 in the former to 51 in the second (Fig. 3a, c). Also RILs DC-84 and DC-72 (macroscopic scores: 3.0 and 3.4, respectively) differed greatly in the number of germlings that were able to penetrate the cell and form micro-colonies: 20.3 micro-colonies/cm^2^ in DC-84, compared to 67.5 in DC-72 (Fig. 3b, d).

At macroscopic level, the micro-colonies evaluated 7 dai differed in appearance: for some RILs, they appeared more floccose than for others with similar score. This is due to different percentage of established micro-colonies able to form conidiophores, as seen in SusBgt_SC_ compared to SusBgt_DC_ (conidiation 20% and less than 2%, respectively (Figs. 3c–f, 4b, c). The results obtained on, for example, SC-83 and SC-106 (Fig. 3c, e) suggest that micro-colony formation and conidiation are not correlated. Even though the SusBgt_DC_ parent allowed very low formation of conidiophores, VxS_DC_ RILs segregated for this trait (Fig. 3f). This suggests that Vada may have (a) gene(s) that promote conidium formation.

### Viability of *Bgt* conidia formed on barley leaves

We tested whether the *Bgt* conidia produced on the nonhost plant barley would be viable, and therefore, able to grow on its natural host, wheat. No fungal growth was observed on the negative controls (not-rubbed with barley leaves) and also not on wheat plants rubbed with Vada-infected leaves (Online Resource 9a–b). This rules out the possibility that any old spores present in the environment or on the surface of leaves would cause the infection. On wheat seedlings treated with SusBgt_DC_ also no colonies of *Bgt* developed (Online Resource 9c), which can be explained by the low conidiation rate observed for this line. Wheat seedlings treated with SusBgt_SC_, SC-45 and DC-02 (Online Resource 9d–f) all produced mildew colonies, indicating that the *Bgt* conidia formed on the leaves of the nonhost barley plants were viable and fit for infecting their natural host. This confirmed that line DC-02 was able to produce viable conidia, while its parents, Vada and SusBgt_DC_, were not or had a very limited production.

### Phenotyping and mapping QTLs for basal resistance to *Bgh*

**Fig. 5:**
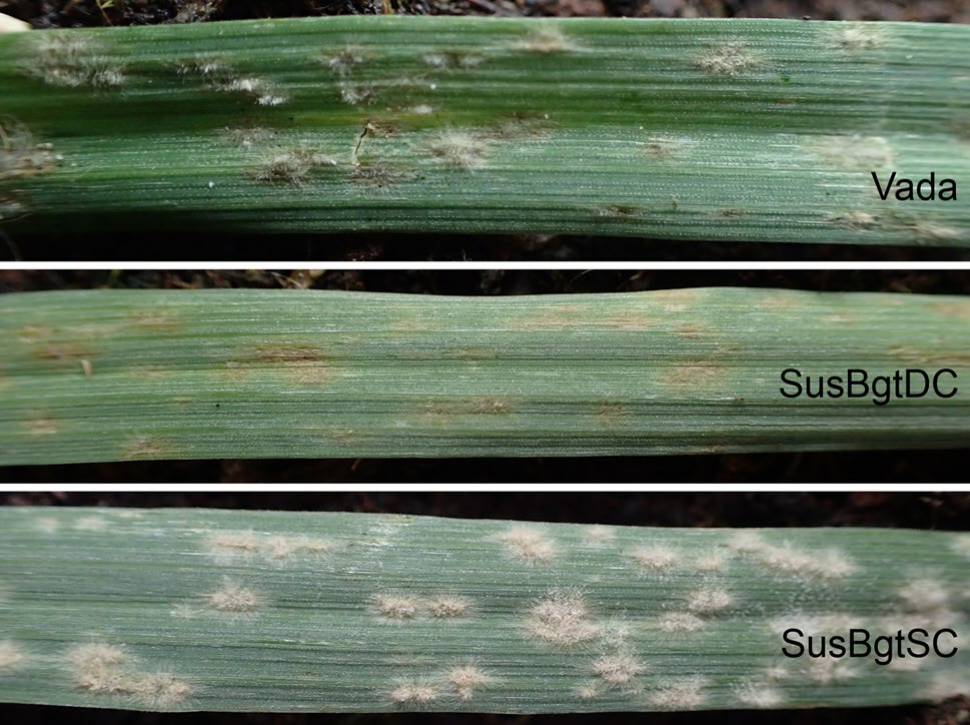
Phenotype of barley (*Hordeum vulgare*) seedlings 7 days after inoculation with the adapted powdery mildew *Blumeria graminis* f.sp. *hordei* (*Bgh*). SusBgt_SC_ (bottom) shows no necrosis and higher infection frequency in comparison to Vada (top) and SusBgt_DC_ (middle); Vada and SusBgt_DC_ show a conspicuous necrotic phenotype

Both mapping populations gave a continuous quantitative distribution for infection frequency (IF), suggesting a polygenic inheritance of the basal resistance to *Bgh*. Transgressive segregation towards resistance and susceptibility was observed, indicating that both parents contributed resistance and susceptibility alleles (Online Resource 10). As early as 4 dai, when seedlings were phenotyped for IF, it was possible to notice the occurrence of HR in some genotypes. We decided then to evaluate also the necrotic reaction, but only after the first inoculation experiment had already been carried out. For this reason, our results regarding necrotic reaction assessment are based on a single inoculation experiment. SusBgt_SC_ showed high IF without macroscopic necrosis, SusBgt_DC_ and Vada a lower level of IF in association with necrosis, which was more obvious on SusBgt_DC_ than on Vada (Fig. 5).

Three QTLs were detected for IF in the two mapping populations, using the average scores from two inoculation experiments (Table 1). QTLs were named ‘*Rbghq*’, standing for ‘Resistance to *Bgh*, quantitative’ and the LOD score of 2.9 was set as threshold. *Rbghq1*, at the telomeric region of the long arm of chromosome 2H, seems to play a major role for *Bgh* resistance in both populations. It is associated with the necrotic phenotype at infection sites. *Rbghq1* has the same peak marker as the nonhost QTL *Rbgnq2*. A second QTL, *Rbghq2*, was mapped for IF and necrosis in the VxS_DC_ population. In the VxS_DC_ population, *Rbghq2* is responsible for a higher percentage of explained phenotypic variance for necrotic reaction than *Rbghq1*, and has SusBgt_DC_ as donor of the resistance allele. In VxS_SC_, HR seems to be mainly governed by *Rbghq1*, with the high LOD score of 26 and accounting for 68% of the explained phenotypic variance. A few additional minor-effect QTLs were detected when the IF data of single experiments for *Bgh* were used (Online Resource 11). One of these minor QTLs, mapped on 4H in the VxS_SC_ population (LOD 3.48), overlaps with the LOD-1 region of the nonhost QTL *Rbgnq3*.

## Discussion

Our study to identify genes that determine nonhost resistance uses natural variation existing among genotypes of a plant species in the level of resistance to a non-adapted pathogen. This is an alternative to the use of interspecific, host × nonhost crosses. Such genetic variation was demonstrated in rice cultivars that, despite being immune to rust fungi, differed in the degree of penetration and haustorium establishment by several cereal rust species (Ayliffe et al. 2011). Other examples include Arabidopsis genotypes for resistance to the bean pathogen *Pseudomonas syringae* pv. *phaseolicola* (Forsyth et al. 2010) and to wheat leaf rust (*P. triticina*) (Shafiei et al. 2007); wheat for resistance to the barley pathogen *Puccinia striiformis* f. sp. *hordei* (Rodrigues et al. 2004); and barley, considered a near-nonhost to several *Puccinia* spp. (Dracatos et al. 2016; Jafary et al. 2008; Yeo et al. 2014). Studies in the barley *Puccinia* pathosystem were made possible by SusPtrit, a barley line developed by accumulation of susceptibility genes or effective selection against resistance genes to the wheat leaf rust (Atienza et al. 2004). SusPtrit is at seedling stage as susceptible to *P. triticina* as susceptible wheat accessions. The SusBgt lines were developed following a similar approach, with *Bgt* as target pathogen (Aghnoum and Niks 2010). The resulting SusBgt lines were by far not as susceptible to *Bgt* as wheat. This indicates the existence of some fixed genes for (this type of) resistance in the barley gene pool, at least as far as represented in the germplasm used by Aghnoum and Niks (2010). The uniformity of such shared genetic factors precludes their identification, and therefore, a large complement of nonhost resistance in the barley *Blumeria* pathosystem remains unresolved. The increased susceptibility status of the SusBgt lines was, nevertheless, sufficient to allow genetic analyses to be performed and part of the genetic components of nonhost resistance to be mapped.

We crossed two SusBgt lines with an immune barley cultivar, Vada, to develop plant material segregating for nonhost resistance to non-adapted *B. graminis* forms. The SusBgt mapping populations allowed, for the first time, identification of QTLs associated with resistance of barley against the non-adapted *Bgt* and *Bghm*. We also evaluated the SusBgt mapping populations for resistance to *Bgh*, enabling comparison between sets of QTLs mapped for nonhost and basal host resistance. The six genomic regions mapped in this study fit into three classes: QTLs mapped for nonhost resistance, for basal host resistance and for both nonhost and host resistance. The set of nonhost resistance QTLs detected in the two populations was almost the same, which can be due to the degree of shared ancestry of the SusBgt lines, which have two parental lines in common (Chame 2 and SusPtrit) and also to the fact that both populations share the resistant parent. However, it could also indicate that there is little variation among barley genotypes in genes causing nonhost resistance to powdery mildew. QTLs effective to *Bgt* were typically also effective to *Bghm*, which can be partly attributed to the close relationship between the pathogens, but also points to a relatively wide spectrum of effectiveness of  genes, simultaneously effective to multiple ff.spp.

Nonhost resistance to haustoria-forming biotrophic fungal pathogens is mostly pre-haustorial but the small percentage of germlings able to form haustoria can be prevented from further developing by plant cell death (Lipka et al. 2005). Most RILs in both mapping populations, as well as Vada, do not show any macroscopic symptom upon *Bgt* and *Bghm* inoculation (Online Resource 4), and microscopy showed that penetration attempts are stopped in association with papillae (Fig. 4a). This is consistent with earlier reports on the papilla-based nature of nonhost resistance of barley to *Blumeria* (Aghnoum and Niks 2010; Trujillo et al. 2004). In some of the RILs that were not immune, infection was associated with very mild necrosis (Fig. 1b). Although RILs were not scored for necrotic reaction to *Bgt* and *Bghm*, we speculate here that *Rbgnq2* might be associated with necrotic reaction in RILs carrying the  susceptibility allele of the largest effect nonhost resistance QTL *Rbgnq1*. *Rbgnq2* has the same map position as *Rbghq1* (Table 1) and we presume that both may represent the same gene. Because *Rbghq1* confers some necrotic reaction to *Bgh*, it may also confer necrosis-associated defence against non-adapted *Bgt* and *Bghm*. *Rbgnq2*/*Rbghq1* may actually represent the powdery mildew resistance gene *MlLa*, which was introgressed into barley cultivars from the barley accession ‘*H. laevigatum*’ and confers an intermediate type of reaction associated with HR phenotype to *Bgh* (Giese et al. 1993; Marcel et al. 2007a). Markers (MWG097, MWG2200) that co-segregated with *MlLa* in the study of Marcel et al. (not published) mapped in the LOD-1 interval of *Rbgnq2*/*Rbghq1.* The parent donor of the *Rbgnq2*/*Rbghq1* resistance allele is Vada, known to carry *MlLa* (Marcel et al. 2007a).

Niks and Marcel (2009) proposed that QTLs represent ‘operative targets’, defined as “host targets that, when manipulated by a pathogen effector, results in enhanced pathogen fitness”. Such operative targets are thought to play a role in plant basal defense responses, and interact with effectors in a minor gene-for-minor gene fashion (Gonzalez et al. 2012; Parlevliet and Zadoks 1977). The ability of a potential pathogen to infect a plant species will mostly rely on its array of effectors and whether they fit the target motifs in the plant. Therefore, failure of non-adapted *B. graminis* species to infect barley can be due to the pathogen lacking appropriate effector molecules and/or due to barley lacking matching operative targets (Niks et al. 2015). As proposed by Antonovics et al. (2013), this would be the consequence of pathogen specialization to its ‘source host’, rather than the result of evolved resistance in the plant. Because barley and wheat evolved from a common ancestor, some barley accessions are expected to still carry variants of operative targets that are compatible with *Bgt* effectors, and such variants may have been accumulated in the SusBgt lines (Aghnoum and Niks 2010). While *Bgt* and *Bghm* manage to partially suppress PTI in the SusBgt lines and establish haustoria, the same is hardly true for *Bghs*, supporting the notion that genes for basal resistance act in a mildew *forma specialis*-specific way (Aghnoum and Niks 2010).

The main determinant of the outcome of barley–*Bgt*/*Bghm* interaction found in this study is *Rbgnq1*. RILs carrying the resistance allele of this QTL are (near) immune to *Bgt* regardless of the background QTLs (Table 2 and Online Resource 6). This is also illustrated by RIL DC-04, segregating for the *Rbgnq1* locus: even though this line carries the susceptibility allele of *Rbgnq2*, *Rbgnq1* seems to be sufficient to confer immunity (Online Resource 8). The high density of markers available, combined with the large effect of *Rbgnq1* made it possible to delimit the gene to a 1.4 cM interval. The phenotypic effect conferred by this gene should be sufficient to allow map-based cloning. The effect size suggests that *Rbgnq1* could rather be called a ‘major gene’. A note of caution is due here, since the immunity of Vada (and barley cultivars, in general) to *Bgt* is the result of the action of several genes regulating an infinity of pathways, and *Rbgnq1* only explains a small part of the spectrum from susceptibility to immunity. Barley lines not carrying *Rbgnq1* (i.e., carrying the SusBgt allele) still have a considerable amount of nonhost resistance left, since no colonies as large as those formed on wheat develop. Despite its large effect on establishment of *Bgt* and *Bghm* on barley, *Rbgnq1* did not reduce IF of the adapted *Bgh*.

Whereas *Rbgnq1* seems a good example of a nonhost resistance gene to which *Bgh* has evolutionary adapted, the above mentioned *Rbgnq2* and the minor QTL *Rbgnq3* have larger effectiveness spectra and appear to confer also basal host resistance to *Bgh*. A QTL mapped for resistance to *Bgh* in the VxS_SC_ population (Online Resource 11) overlaps with the LOD-1 region of *Rbgnq3*. Surprisingly, the resistance allele of *Rbgnq3* is contributed by the susceptible parent, suggesting that the SusBgt lines have at least one resistance factor that lacks in Vada. At a similar position on chromosome 4H, Jafary et al. (2008) reported the mapping of a QTL effective to four non-adapted rust species, also with the susceptible parent (SusPtrit) contributing the resistance allele. This chromosomal region is, therefore, associated with a wide-spectrum resistance against different fungal pathogens. Association of this region with resistance to non-adapted powdery mildews and rusts can be due to the presence of many linked resistance genes or to the same gene. Resistance to several fungal pathogens caused by a single gene has been reported in wheat, for the genes *Lr34* (synonyms: *Yr18*/*Sr57*/*Pm38*), *Lr67* (*Yr46*/*Sr55*/*Pm46*) and *Lr46* (*Yr29*/*Sr58*/*Pm39*). These three broad-spectrum resistance genes are effective against all tested races of the wheat leaf rust, stem rust and stripe rust fungi (*P. triticina*, *P. striiformis* f.sp. *tritici* and *P. graminis* f.sp. *tritici*, respectively) and also the wheat powdery mildew fungus *Bgt* (Ellis et al. 2014; Herrera-Foessel et al. 2014; Kolmer et al. 2008). Two of these genes, *Lr34* and *Lr67*, have been cloned and found to encode membrane-localized transporter proteins (Krattinger et al. 2009; Moore et al. 2015).

Histological studies are helpful to elucidate certain aspects of the interaction, like the rate of haustorium formation and conidiation. Different numbers of established micro-colonies were found for RILs showing similar macroscopic scores. This is probably due to different sizes of micro-colonies in different RILs: some RILs may allow more secondary hyphal growth than others or even formation of some conidiophores. This might be caused by variation in gene(s) acting at post-invasion nonhost resistance. We also observed that haustorium establishment is not always associated with successful conidiophore formation (Fig. 3), in agreement with previous reports by Aghnoum and Niks (2010) that barley lines showing similar levels of haustorium establishment by non-adapted *B. graminis* forms differed in conidiation rates. This indicates that several layers of defence are involved in basal defence, acting at different stages of pathogen development. Conidiation segregated among RILs from VxS_DC_, even though SusBgt_DC_ had a conidiation rate close to zero (Fig. 3f). This suggests that the immune Vada carries, underneath a very effective pre-haustorial defence, some factors that would allow the pathogen to further develop and complete its life cycle. Due to the limited number of RILs that actually allowed some degree of *Bgt* growth, it was not possible to map the QTL(s) determining conidiation.

Our work is analogous to that of Jafary et al. (2006, 2008), who mapped QTLs for nonhost resistance to non-adapted rust species in three barley mapping populations. Rusts and powdery mildews are both obligate biotrophs, and nonhost and basal resistance in these two pathosystems are typically pre-haustorial (Niks 1986; Olesen et al. 2003). A high diversity of loci was implicated in resistance to rusts, and immunity in different tested barley accessions was shown to be due to different combinations of genes (Jafary et al. 2008). Some QTLs mapped in the rust study were species-specific, others were effective to more than one rust fungal species. Our results also demonstrate polygenic inheritance for nonhost resistance to *Bgt* and *Bghm*, but because we only used Vada as immune parent, it still remains to be investigated how wide diversity there is to protect barley against non-adapted powdery mildews. Loci mapped for nonhost and basal host resistance to rusts were found to be significantly associated with loci for plant defence gene homologs (Jafary et al. 2008) such as peroxidases (Gonzalez et al. 2010), in agreement with the hypothesis that these two types of resistance rely on similar principles (Aghnoum and Niks 2010; Jafary et al. 2006; Marcel et al. 2007b; Schweizer and Stein 2011). In the present study at least two QTLs are in common for non-adapted and adapted mildew forms (*Rbgnq3* and *Rbgnq2*/*Rbghq1*), also pointing to an overlap on genetic mechanisms mediating nonhost and basal host resistance. The indication that *MlLa* is not only effective to *Bgh* but also against non-adapted mildews is an interesting finding, with no parallel in the barley rust pathosystem.

This research extends our knowledge on the genetic basis of nonhost resistance. We confirmed the polygenic mode of inheritance in barley to powdery mildew and that plant genetic factors determining establishment by haustorium formation act independently from factors determining level of conidiation. Fine-mapping and complementation studies are necessary to isolate the underlying genes for nonhost resistance to powdery mildew. Types of genes expected to be found may belong to an as diverse array of gene families as found for basal/quantitative host resistance, rather than to one family, as accepted for race specific hypersensitive resistance (Lee and Yeom 2015). In a parallel study, our group is close to cloning the gene responsible for *Rbgnq1* resistance. Fine-mapping resulted in a QTL interval comprising 20 candidate genes (Romero et al. unpublished). The cloning of nonhost resistance gene(s) in barley will open up the possibility of transferring this resistance to wheat, where its orthologues are likely to be suppressed by *Bgt* effectors (Douchkov et al. 2014). There are several examples demonstrating successful transfer of nonhost resistance across species (Du et al. 2015; Johnston et al. 2013; Lacombe et al. 2010; Lee et al. 2016, 2017). The QTLs mapped in this study could, in the future, emerge as a valuable resource for Triticeae disease resistance breeding programs.

### Author contribution statement

REN, CCTR, and JPV designed the research. AV carried out the single seed descent procedure to develop the mapping populations and assisted on the set up of the experiments. CCTR and JPV performed the QTL mapping experiments. AH conducted the genotyping-by-sequencing experiments. MM analysed the genotyping data and prepared the high-density genetic maps. CCTR, REN, JPV, and MM contributed to data analysis and interpretation. Manuscript was written by CCTR, REN and MM.

## Electronic supplementary material

Below is the link to the electronic supplementary material.

Online Resource 1 QTL mapping files for Vada × SusBgt_SC_ and Vada × SusBgt_DC_ mapping populations (XLSX 356 kb)

Online Resource 2 Macroscopic phenotypes of parental barley (*Hordeum vulgare*) lines Vada, SusBgt_SC_ and SusBgt_DC_ upon inoculation with different ff.spp. of *Blumeria graminis*. (a) f.sp. *hordei*-*secalini* (*Bghs*), the pathogen of *H. secalinum*, 14 days after inoculation (dai): no macroscopically visible symptoms on the surface of the leaves. Development of micro-colonies is observed on the SusBgt lines 7 dai with (b) f.sp. *tritici* (*Bgt*), the pathogen of wheat and (c) f.sp. *hordei*-*murini* (*Bghm*), the pathogen of *H. murinum* (PDF 84 kb)

Online Resource 3 (DOCX 13 kb)

Online Resource 4 (DOCX 22 kb)

Online Resource 5 High-density genetic maps of Vada × SusBgt_SC_ and Vada × SusBgt_DC_ mapping populations (XLSX 5984 kb)

Online Resource 6 (DOCX 14 kb)

Online Resource 7 (XLSX 30 kb)

Online Resource 8 Progeny of RIL DC-04, 7 days after inoculation (dai) with *Blumeria graminis* f.sp. *tritici* (*Bgt*). Seedlings on the top and middle show a resistant phenotype while the one at the bottom of the picture shows a susceptible phenotype (PDF 107 kb)

Online Resource 9 (DOCX 1949 kb)

Online Resource 10 (DOCX 17 kb)

Online Resource 11 (DOCX 15 kb)
