# Supplementary material for: Mapping resistance to powdery mildew in barley reveals a large-effect nonhost resistance QTL
Source: Theor Appl Genet. 2018 Jan 25;131(5):1031–45. doi: 10.1007/s00122-018-3055-0 (PMC5895680; doi:10.1007/s00122-018-3055-0)

Article title: Mapping Resistance to Powdery Mildew in Barley Reveals a Large-Effect Nonhost Resistance QTL

Authors: Cynara C. T. Romero, Jasper P. Vermeulen, Anton Vels, Axel Himmelbach, Martin Mascher and Rients E. Niks

Author for correspondence: Rients E. Niks, Wageningen University and Research

Email: rients.niks@wur.nl

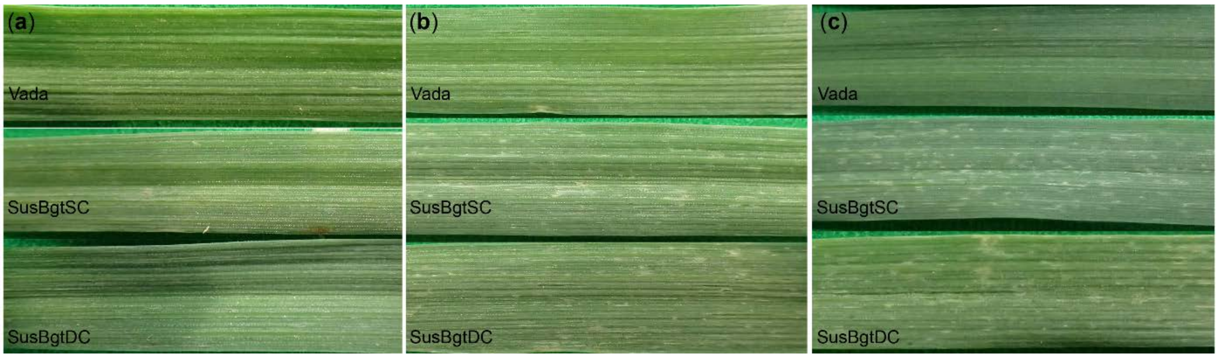

Supplement: Supplementary file 2 — Online Resource 2 Macroscopic phenotypes of parental barley (Hordeum vulgare) lines Vada, SusBgtSC and SusBgtDC upon inoculation with different ff.spp. of Blumeria graminis. (a) f.sp. hordei-secalini (Bghs), the pathogen of H. secalinum, 14 days after inoculation (dai): no macroscopically visible symptoms on the surface of the leaves. Development of micro-colonies is observed on the SusBgt lines 7 dai with (b) f.sp. tritici (Bgt), the pathogen of wheat and (c) f.sp. hordei-murini (Bghm), the pathogen of H. murinum (PDF 84 kb) [file 122_2018_3055_MOESM2_ESM.pdf]
