# Supplementary material for: Mapping resistance to powdery mildew in barley reveals a large-effect nonhost resistance QTL
Source: Theor Appl Genet. 2018 Jan 25;131(5):1031–45. doi: 10.1007/s00122-018-3055-0 (PMC5895680; doi:10.1007/s00122-018-3055-0)
Supplement: Supplementary file 3 — Online Resource 3 (DOCX 13 kb) [file 122_2018_3055_MOESM3_ESM.docx]

Article title: Mapping Resistance to Powdery Mildew in Barley Reveals a Large-Effect Nonhost Resistance QTL

Authors: Cynara C. T. Romero, Jasper P. Vermeulen, Anton Vels, Axel Himmelbach, Martin Mascher and Rients E. Niks

Author for correspondence: Rients E. Niks, Wageningen University and Research

Email: rients.niks@wur.nl

Summary of data for the inoculation experiments with *Blumeria graminis* f.sp. *tritici* (*Bgt*) and f.sp. *hordei-murini* (*Bghm*) for the two SusBgt mapping populations.

|  | Mapping population | | | | |
| --- | --- | --- | --- | --- | --- |
|  | Vada x SusBgt_SC_ | |  | Vada x SusBgt_DC_ | |
| *Formae specialis* | *Bgt* | *Bghm* |  | *Bgt* | *Bghm* |
| Average inoculation density (conidia/mm^2^) | 28.3 | 21.1 |  | 23.3 | 23.7 |
| Correlation between reps | 0.83 | 0.69 |  | 0.84 | 0.81 |
| % RILs with score below 2 | 75.2% | 79.1% |  | 76.5% | 77.9% |
| % RILs with score above 4 | 6.4% | 1.8% |  | 8.7% | 5.3% |
