# Supplementary material for: Mapping resistance to powdery mildew in barley reveals a large-effect nonhost resistance QTL
Source: Theor Appl Genet. 2018 Jan 25;131(5):1031–45. doi: 10.1007/s00122-018-3055-0 (PMC5895680; doi:10.1007/s00122-018-3055-0)
Supplement: Supplementary file 4 — Online Resource 4 (DOCX 22 kb) [file 122_2018_3055_MOESM4_ESM.docx]

Article title: Mapping Resistance to Powdery Mildew in Barley Reveals a Large-Effect Nonhost Resistance QTL

Authors: Cynara C. T. Romero, Jasper P. Vermeulen, Anton Vels, Axel Himmelbach, Martin Mascher and Rients E. Niks

Author for correspondence: Rients E. Niks, Wageningen University and Research

Email: rients.niks@wur.nl

Frequency distributions of the macroscopic disease scores in two barley mapping populations inoculated with *Blumeria* *graminis* f.sp. *tritici* (*Bgt*) and f.sp *hordei-murini* (*Bghm*). Values on the y-axis show the number of RILs, and the x-axis represent the classes of disease scores from 1 to 5, or larger than 5. (a) VxS_SC_ population inoculated wth *Bgt*, (b) the VxS_DC_ population inoculated with *Bgt*. (c) VxS_SC_ population inoculated wth *Bghm* and (d) VxS_DC_ population inoculated with *Bghm*.
