# Supplementary material for: Mapping resistance to powdery mildew in barley reveals a large-effect nonhost resistance QTL
Source: Theor Appl Genet. 2018 Jan 25;131(5):1031–45. doi: 10.1007/s00122-018-3055-0 (PMC5895680; doi:10.1007/s00122-018-3055-0)
Supplement: Supplementary file 6 — Online Resource 6 (DOCX 14 kb) [file 122_2018_3055_MOESM6_ESM.docx]

Article title: Mapping Resistance to Powdery Mildew in Barley Reveals a Large-Effect Nonhost Resistance QTL

Authors: Cynara C. T. Romero, Jasper P. Vermeulen, Anton Vels, Axel Himmelbach, Martin Mascher and Rients E. Niks

Author for correspondence: Rients E. Niks, Wageningen University and Research

Email: rients.niks@wur.nl

Average macroscopic infection scores for VxS_DC_ recombinant inbred lines (RILs) grouped according to presence (+) or absence (-) of the resistance allele of QTLs mapped for *Blumeria graminis* f.sp. *tritici* (*Bgt*) and f.sp *hordei-murini* (*Bghm*). Resistance alleles of all QTLs are from Vada (V). Values in each column that share the same letter are not significantly different (*P* < 0.05).

| *Rbgnq1* (V) | *Rbgnq2* (V) | *Rbgnq4* (V) | Number of RILs* | *Bgt* |  | *Bghm* |  |
| --- | --- | --- | --- | --- | --- | --- | --- |
| **+** | **+** | **+** | 21 | 1.1 | a | 1.1 | a |
| **+** | **+** | **-** | 10 | 1.1 | ab | 1.5 | ab |
| **+** | **-** | **+** | 15 | 1.2 | ab | 1.1 | a |
| **+** | **-** | **-** | 14 | 1.1 | ab | 1.2 | ab |
| **-** | **+** | **+** | 7 | 1.8 | ab | 1.5 | ab |
| **-** | **+** | **-** | 14 | 1.8 | b | 2.0 | b |
| **-** | **-** | **+** | 8 | 3.9 | c | 1.4 | ab |
| **-** | **-** | **-** | 12 | 3.3 | c | 3.3 | c |

^*^ Total number of RILs analysed: 101 for *Bgt* and 99 for *Bghm*. The number of RILs in each group differed slightly (±1 RIL) between *Bgt* and *Bghm* scores because of missing phenotyping data. Ten RILs were excluded from the analysis because there was a recombination point close to the peak markers of *Rbgnq1*.
