# Supplementary material for: Mapping resistance to powdery mildew in barley reveals a large-effect nonhost resistance QTL
Source: Theor Appl Genet. 2018 Jan 25;131(5):1031–45. doi: 10.1007/s00122-018-3055-0 (PMC5895680; doi:10.1007/s00122-018-3055-0)

Article title: Mapping Resistance to Powdery Mildew in Barley Reveals a Large-Effect Nonhost Resistance QTL

Authors: Cynara C. T. Romero, Jasper P. Vermeulen, Anton Vels, Axel Himmelbach, Martin Mascher and Rients E. Niks

Author for correspondence: Rients E. Niks, Wageningen University and Research

Email: rients.niks@wur.nl

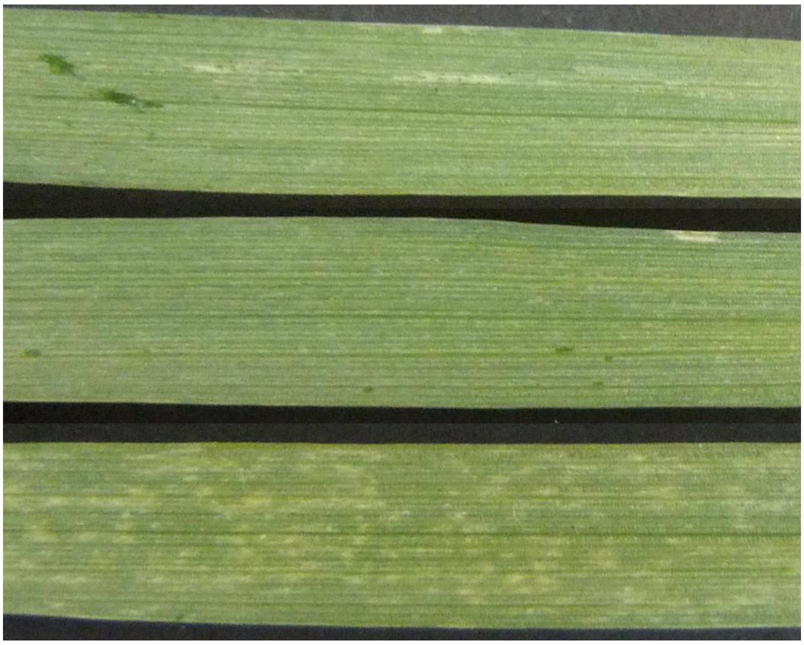

Supplement: Supplementary file 8 — Online Resource 8 Progeny of RIL DC-04, 7 days after inoculation (dai) with Blumeria graminis f.sp. tritici (Bgt). Seedlings on the top and middle show a resistant phenotype while the one at the bottom of the picture shows a susceptible phenotype (PDF 107 kb) [file 122_2018_3055_MOESM8_ESM.pdf]
