# Supplementary material for: Mapping resistance to powdery mildew in barley reveals a large-effect nonhost resistance QTL
Source: Theor Appl Genet. 2018 Jan 25;131(5):1031–45. doi: 10.1007/s00122-018-3055-0 (PMC5895680; doi:10.1007/s00122-018-3055-0)
Supplement: Supplementary file 9 — Online Resource 9 (DOCX 1949 kb) [file 122_2018_3055_MOESM9_ESM.docx]

Article title: Mapping Resistance to Powdery Mildew in Barley Reveals a Large-Effect Nonhost Resistance QTL

Authors: Cynara C. T. Romero, Jasper P. Vermeulen, Anton Vels, Axel Himmelbach, Martin Mascher and Rients E. Niks

Author for correspondence: Rients E. Niks, Wageningen University and Research

Email: rients.niks@wur.nl

Viability test of *Blumeria graminis* f.sp. *tritici* (*Bgt*) conidia produced on barley epidermis: wheat leaves 5 days after being in direct contact with barley leaves of different genotypes inoculated with *Bgt*; (a) Negative control; (b) Vada; (c) SusBgt_DC_; (d) SusBgt_SC_; (e) SC-45 and (f) DC-02.

| (a) Negative control | 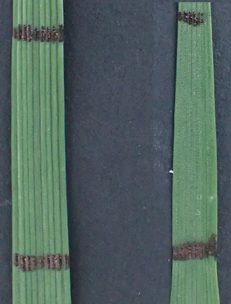 | 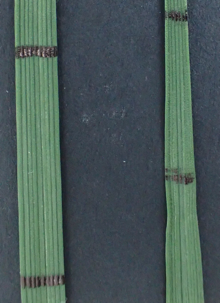 | 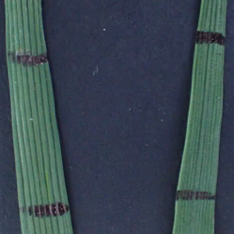 |
| --- | --- | --- | --- |
| (b) Vada | 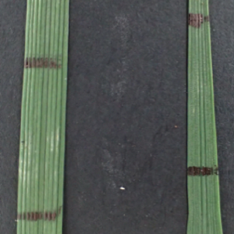 | 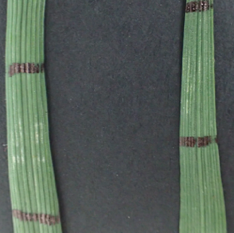 | 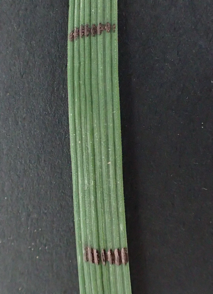 |
| (c) SusBgt_DC_ | 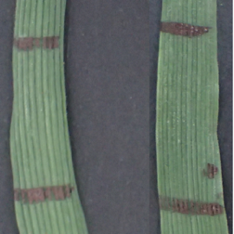 | 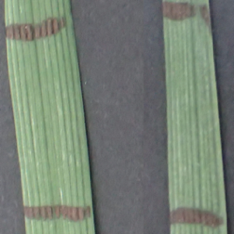 | 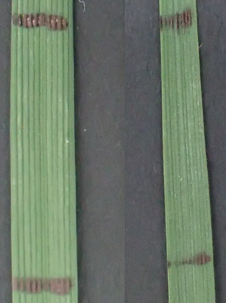 |
| (d) SusBgt_SC_ | 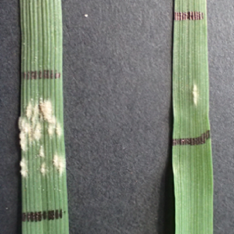 | 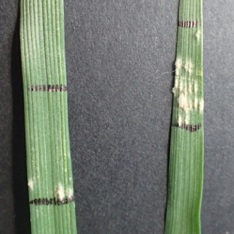 | 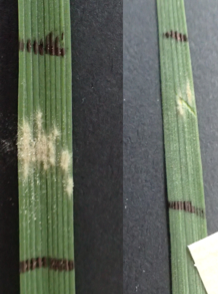 |
| (e) SC-45 | 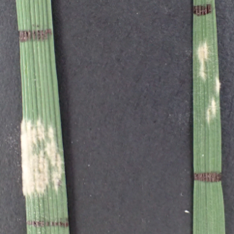 | 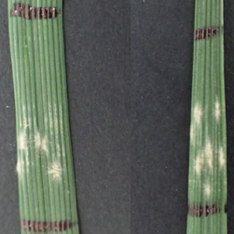 | 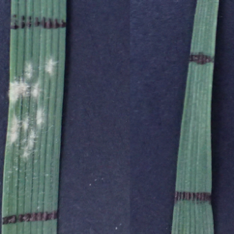 |
| (f) DC-02 | 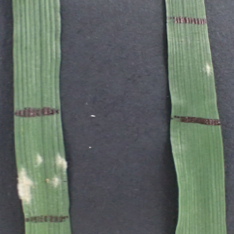 | 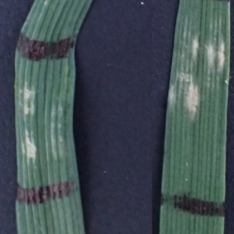 | 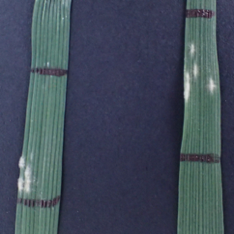 |
