# Supplementary material for: Mapping resistance to powdery mildew in barley reveals a large-effect nonhost resistance QTL
Source: Theor Appl Genet. 2018 Jan 25;131(5):1031–45. doi: 10.1007/s00122-018-3055-0 (PMC5895680; doi:10.1007/s00122-018-3055-0)
Supplement: Supplementary file 10 — Online Resource 10 (DOCX 17 kb) [file 122_2018_3055_MOESM10_ESM.docx]

Article title: Mapping Resistance to Powdery Mildew in Barley Reveals a Large-Effect Nonhost Resistance QTL

Authors: Cynara C. T. Romero, Jasper P. Vermeulen, Anton Vels, Axel Himmelbach, Martin Mascher and Rients E. Niks

Author for correspondence: Rients E. Niks, Wageningen University and Research

Email: rients.niks@wur.nl

Frequency distributions of the macroscopic disease scores in the SusBgt populations inoculated with *Blumeria graminis* f.sp. *hordei* (*Bgh*). Values on the y-axis show the number of Recombinant Inbred Lines (RILs), and the x-axis represent average infection frequency (IF) classes (a) Vada x SusBgt_SC_ (b) Vada x SusBgt_DC_.
