# Supplementary material for: Mapping resistance to powdery mildew in barley reveals a large-effect nonhost resistance QTL
Source: Theor Appl Genet. 2018 Jan 25;131(5):1031–45. doi: 10.1007/s00122-018-3055-0 (PMC5895680; doi:10.1007/s00122-018-3055-0)
Supplement: Supplementary file 11 — Online Resource 11 (DOCX 15 kb) [file 122_2018_3055_MOESM11_ESM.docx]

Article title: Mapping Resistance to Powdery Mildew in Barley Reveals a Large-Effect Nonhost Resistance QTL

Authors: Cynara C. T. Romero, Jasper P. Vermeulen, Anton Vels, Axel Himmelbach, Martin Mascher and Rients E. Niks

Author for correspondence: Rients E. Niks, Wageningen University and Research

Email: rients.niks@wur.nl

QTL mapping results for resistance to *Blumeria graminis* f.sp. *hordei* (*Bgh*) for all inoculation experiments.

| Mapping  population | Rep^a^ | Trait^b^ | QTL name^c^ | Peak marker | Chr^d^ | Position | LOD | % Expl^e^ | Additive^f^ | Donor^g^ | Mapped for NHR?^h^ |
| --- | --- | --- | --- | --- | --- | --- | --- | --- | --- | --- | --- |
|  | Rep1 | IF | *Rbghq1* | SC-C2_SNP54 | 2H | 153.9 | 10.97 | 37.1 | -6.069 | Vada | *Bgt, Bghm* |
|  | Rep1 | Nec | *Rbghq1* | SC-C2_SNP54 | 2H | 153.9 | 33.02 | 75.6 | 1.00 | Vada |  |
|  | Rep1 | Nec |  | SC-C7-SNP24 | 7H | 64.0 | 3.12 | 3.5 | 0.217 | Vada |  |
|  | Rep2 | IF |  | SC-C2_SNP9 | 2H | 23.6 | 3.10 | 10.2 | 2.511 | SusBgtSC |  |
| VxS_SC_ | Rep2 | IF | *Rbghq1* | SC-C2_SNP54 | 2H | 153.9 | 8.12 | 24.1 | -3.766 | Vada | *Bgt, Bghm* |
|  | Rep2 | IF |  | SC-C4_SNP17 | 4H | 41.9 | 3.48 | 9.3 | 2.346 | SusBgtSC | *Bghm* |
|  | Rep2 | IF | *Rbghq3* | SC-C6_SNP41 | 6H | 119.2 | 3.87 | 10.4 | -2.494 | Vada |  |
|  | Rep2 | Nec | *Rbghq1* | SC-C2_SNP54 | 2H | 153.9 | 26.01 | 68 | 1.084 | Vada |  |
|  | Average | IF | *Rbghq1* | SC-C2_SNP54 | 2H | 153.9 | 11.62 | 35.1 | -4.795 | Vada | *Bgt, Bghm* |
|  | Average | IF | *Rbghq3* | SC-C6_SNP40 | 6H | 116.8 | 2.87 | 7.1 | -2.208 | Vada |  |
|  | Rep1 | IF |  | DC-C1_SNP39 | 1H | 108.9 | 3.04 | 7.8 | 1.571 | SusBgtDC |  |
|  | Rep1 | IF | *Rbghq1* | DC-C2_SNP58 | 2H | 159.7 | 4.78 | 14.5 | -2.084 | Vada | *Bgt, Bghm* |
|  | Rep1 | IF | *Rbghq2* | DC-C7_SNP1 | 7H | 0.0 | 6.9 | 21.5 | 2.572 | SusBgtDC |  |
|  | Rep2 | IF | *Rbghq1* | DC-C2_SNP58 | 2H | 159.7 | 5.63 | 15.2 | -3.224 | Vada | *Bgt, Bghm* |
| VxS_DC_ | Rep2 | IF | *Rbghq2* | DC-C7_SNP2 | 7H | 3.2 | 9.88 | 29.2 | 4.494 | SusBgtDC |  |
|  | Rep2 | Nec | *Rbghq1* | DC-C2_SNP58 | 2H | 159.7 | 4.02 | 9.8 | 0.380 | Vada | *Bgt, Bghm* |
|  | Rep2 | Nec | *Rbghq2* | DC-C7_SNP1 | 7H | 0.0 | 13.24 | 39.4 | -0.770 | SusBgtDC |  |
|  | Average | IF | *Rbghq1* | DC-C2_SNP58 | 2H | 159.7 | 8.32 | 20.6 | -2.738 | Vada | *Bgt, Bghm* |
|  | Average | IF | *Rbghq2* | DC-C7_SNP2 | 7H | 3.2 | 11.82 | 31.6 | 3.406 | SusBgtDC |  |

**^a^** The inoculation experiment from which the data was taken for QTL analysis, whether a single experiment from replicate 1, 2, or the average of both inoculation experiments;

**^b^**  Trait analysed, whether infection frequency (IF) or necrosis (Nec);

**^c^** QTLs were named only when mapped for the average of inoculation experiments;

**^d^** The chromosome (linkage group) in which the QTL was mapped;
**^e^** The proportion of phenotypic variance explained by the QTL;

**^f^** The effect of having an allele from Vada on the macroscopic infection score;

**^g^** Parent donor of resistance allele

**^h^** Indicates whether the QTL was mapped for nonhost resistance
